# Interplay between Cellular and Molecular Mechanisms Underlying Inflammatory Bowel Diseases Development—A Focus on Ulcerative Colitis

**DOI:** 10.3390/cells9071647

**Published:** 2020-07-09

**Authors:** Iuliana Samoilă, Sorina Dinescu, Marieta Costache

**Affiliations:** 1 Department of Biochemistry and Molecular Biology, University of Bucharest, 050095 Bucharest, Romania; iuliana.samoila@bio.unibuc.ro (I.S.); marieta.costache@bio.unibuc.ro (M.C.); 2 Research Institute of the University of Bucharest, 050663 Bucharest, Romania

**Keywords:** inflammasome, NLRP3, autophagy, miRNAs in IBD, inflammatory bowel diseases

## Abstract

Inflammatory bowel diseases (IBD) are defined by the continuous inflammation of the gastrointestinal tract. During inflammation, the number of pathogens in the intestinal epithelium increases, leading to inflammasome assembly. Inflammasome activation is meant to protect the intestinal epithelial barrier from further damage by maintaining homeostasis. Although its purpose is to protect the cells, excessive nucleotide-binding oligomerization domain-like receptor and pyrin domain-containing protein 3 (NLRP3) inflammasome assembly is responsible for the synthesis of a high number of pro-inflammatory cytokines. The activation of two crucial pathways, autophagy process, and unfolded protein response, is initiated for restoring homeostasis. Aberrant expression of miRNAs and lncRNAs also interfere with the pathogenic mechanisms of IBD, as these non-coding transcripts play key roles in regulation of biological processes, such as inflammation and immunity. This review thoroughly describes the cellular and molecular mechanism that trigger and perpetuate inflammation in ulcerative colitis (UC) patients.

## 1. Introduction

Inflammatory bowel diseases (IBD) are characterized by chronic inflammation of the gastrointestinal tract, with alternating phases of clinical relapse and remission [1]. The cause of IBD is still unknown, although it is believed to be a combination of multiple environmental factors, such as stress, diet, along with the genetic inheritance patterns, all leading to an excessive and abnormal immune response against commensal gut flora [2,3,4].

Under normal conditions, the immune system is responsible for preventing the overwhelming amounts of harmful bacteria that could enter the lamina propria, whilst tolerating the commensal bacteria. Disturbance in the microbiota results in growing populations of harmful bacteria in the intestine, which directly affects the intestinal epithelial cells (IECs) [5]. A damaged mucus layer causes physical contact between IECs and bacteria in the mucosa, resulting in high luminal antigen uptake and a massive immune response. The antigens activate innate immune cells—macrophages (MPs) and dendritic cells (DCs), through pattern recognition receptors (PRRs). The two cell types activate nuclear factor kappa B (NF-kB) signaling pathway, resulting in pro-inflammatory cytokine production, tumor necrosis factor α (TNF-α), interleukin (IL)-6, IL-12, IL-23, and IL-1β. Subsequent, MPs and DCs present the antigens to naïve T cells to stimulate their differentiation into Th2 cells, which along with natural killer T cells, have the ability to produce IL-13, damaging the intestinal epithelial barrier by altering the protein composition of tight junctions of epithelial cells. Injured IECs release IL-37, exerting anti-inflammatory effects, by reducing TNF-α and IL-1β production from the lamina propria [6].

PRRs are critical components in regulating the aberrant innate immune responses in the intestine. One of the most important PRRs for IBD pathogenesis is nucleotide-binding oligomerization domain-like receptor and pyrin domain-containing protein 3 (NLRP3), as it rapidly emerges in the assembly of an inflammasome complex, under a range of stimuli. The inflammasome complex is assembled once caspase-1 is activated and IL-1β and IL-18 are synthesized [7].

Moreover, numerous studies have correlated the interaction between two fundamental biologic pathways in inflammatory ulcerative colitis (UC) pathogenesis. The first one is represented by the endoplasmic reticulum stress (ERS), which is characterized by a great accumulation of incorrect folded protein in the ER. The second one is represented by the autophagy process, which regulates the removal of protein aggregates and invading antigens [8]. By degrading intracellular pathogens and microbial toxins in the intestine, autophagy promotes cell survival, including IECs. If autophagy process is unbalanced, IECs function could be altered; hence, disruption of intestinal barrier integrity, ERS could be activated, and reactive oxygen species (ROS) production exacerbated, all leading to UC pathogenesis [9].

Alongside with ER and autophagy, some non-coding RNA transcripts are also involved in the regulation of many biological processes, including cellular proliferation and maturation, as well as in the induction of chronic inflammation in IBD patients. Although the information is mainly focused on their potential to be diagnosis biomarkers, researchers have recently focused on these small molecules’ therapeutic capability [10,11].

This review aims to thoroughly characterize the cellular and molecular mechanisms that either trigger or perpetuate the inflamed condition of the colon. The center of the attention in this manuscript revolves around autophagy, ERS and inflammation, as well as non-coding RNA transcripts in the IBD, with a focus on UC pathology.

## 2. Inflammasomes

Innate immunity is considered to be the first line defense, which can differentiate between pathogenic microbes and normal host molecules or commensal gut flora [12]. The innate immune system can be triggered by a variety of stimuli, such as exogenous microbes or endogenous danger signals, via innate immune sensors PRRs—toll-like receptors (TLRs) and nod-like receptors (NLRs). These PRRs are expressed by cells, including MPs, DCs or even epithelial cells, and can recognize a wide variety of stimuli, such as damage-associated molecular patterns (DAMPs) and pathogen-associated molecular patterns (PAMPs) [7,13]. When these stimuli are recognized, a cytosolic multi-protein signaling complex, called “inflammasome”, is activated. This structure is able to control and mediate the host immune responses, protecting the organism from the invasion of pathogens. Many studies that focused on inflammasomes’ molecular mechanisms reported that they can be associated with inflammation- and immune-related disorders, such as diabetes, atherosclerosis or IBD [14]. A number of inflammasomes has been described, including NLRP1, NLRP3, Absent in Melanoma 2 (AIM2) and Pyrin, among which NLRP3 being thoroughly characterized.

### 2.1. Molecular Mechanisms of NLRP3 Inflammasome Signaling

NLRs are multidomain proteins with a central nucleotide-binding and oligomerization domain (NACHT), C-terminal leucine-rich repeats (LRRs) and N-terminal caspase recruitment (CARD) or pyrin (PYD) domains. Usually, NACHT and LRRs domains connection is inhibited, preventing inflammasome formation [12]. Three components form the NLRP3 inflammasome—NLRP3 protein, adapter protein apoptosis-associated speck-like protein (ASC) and procaspase-1 [13]. NLRP3 inflammasome has been divided into canonical and non-canonical activation pathways.

Activation of canonical NLRP3 inflammasome is a two-step process, namely “priming” and “activation”. During the “priming” step, under stress conditions, DAMPs and PAMPs are being recognized by PRRs, leading to NF-kB signaling activation. Activated NF-kB increases transcription and translation of immature pro-forms of cytokines pro-IL-1β and pro-IL-18. The second step involves inflammasome formation and activation, which is triggered by a variety of stimuli, such as bacterial, viral, or fungal pathogens, extracellular adenosine triphosphate (ATP) and ROS. These stimuli promote a cascade of events, in order to secrete these pro-inflammatory cytokines into the extracellular space. NLRP3 protein interacts with ASC, which later on will be conjugated with procaspase-1 for inflammasome formation. Once the inflammasome is assembled, procaspase-1 is self-cleaved into its active form, caspase-1 (CAS-1). Activated CAS-1 will promote maturation of pro-inflammatory cytokines pro-IL-1β and pro-IL-18 into their active form, respectively IL-1β, and IL-18, and also cleavage of gasdermin D, which helps IL-1β and IL-18 be secreted out of MPs through plasma membrane pores [13,14,15]. 

The non-canonical NLRP3 inflammasome pathway can be activated by enteric bacteria, such as *Escherichia coli*, *Vibrio cholera*, or *Citrobacter rodentium*. In this case, another caspase is required for CAS-1 activation in MPs infected with Gram-negative bacteria. The most important caspase that interacts and senses the stimuli is caspase-11, a murine inflammatory caspase, whereas the human analogs are represented by caspase-4 and caspase-5. Once these stimuli are detected, activation of the NLRP3 inflammasome is activated, together with the secretion of IL-1β and IL-18. It has also been postulated that the activation of the non-canonical inflammasome pathway might be correlated with potassium (K^+^) efflux [13,16]. Most of the NLRP3 activation stimuli, especially ATP, disturb the permeability of macrophages’ membrane to K^+^, leading to a decreased intracellular K^+^ concentration. As cytosolic K^+^ decreases, the concentration of K^+^ efflux increases [17]. It is known that a low concentration of K^+^ is one of the key requirements for NLRP3 inflammasome activation, although it remains insufficiently studied whether K^+^ efflux alone is enough to trigger activation of the NLRP3 inflammasome, or if it is just one of the many signals involved in this process [18].

### 2.2. NLRP3 Inflammasome Activation in Ulcerative Colitis

**Figure 1:**
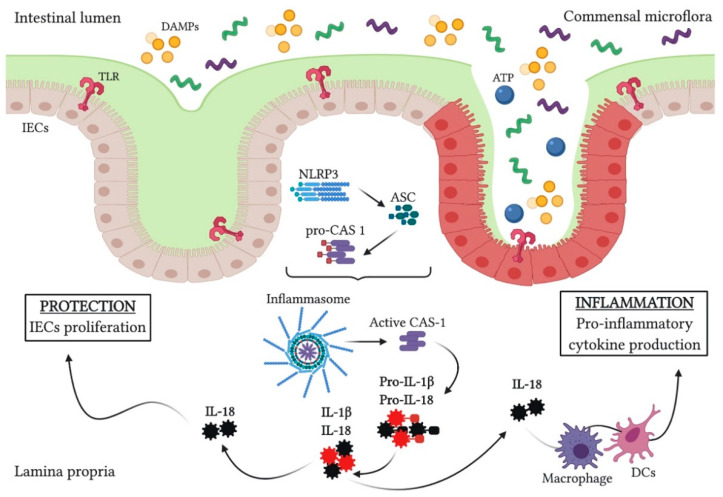
NLRP3 inflammasome activation can have protective and inflammatory effects in the intestinal epithelium. DAMPs and ATP molecules are recognized by TLRs on the IECs, which results in NLRP3 protein recruits adaptor protein ASC. This complex recruits pro-caspase 1, leading to inflammasome assembly and caspase-1 activation. Further on, activated caspase-1 promotes activation of pro-inflammatory cytokines IL-1β and IL-18. IL-18 is necessary for IECs proliferation, but excessive production of IL-18 leads to activation of immune cells and overproduction of pro-inflammatory cytokines, promoting inflammation.

As PRRs recognize DAMPs and PAMPs and CAS-1 is activated, NLRP3 inflammasome starts assembly, which leads to excessively production of IL-1β and IL-18 cytokines by epithelial cells—Paneth cells or antigen-presenting cells. A high number of these cytokines is associated with inflammation, by engaging and activating immune cells, as well as promoting pro-inflammatory cytokines and chemokines production [14,19]. Consequently, an increased level of IL-1β and IL-18, as well as CAS-1, has been identified in the inflamed mucosa, respectively in the MPs and intestinal tissue of UC patients. Studies regarding the absence of *Cas-1* in mice with induced colitis are split between two theories. First theory suggests that mice treated with Pralnacasan, a CAS-1 inhibitor, or with IL-1R antagonist experienced a significantly less severe colitis, due to a lower expression of IL-1β and IL-18 [20,21]. On the other hand, the second and more recently postulated theory, demonstrated that *Cas-1*^−/−^ mice had an even more aggravated colitis, probably due to the insufficiency of IL-18, which is an early trigger of tissue repair [21,22]. Taking into account the aforementioned theories, researchers believe that inflammasome activation response is dependent on the normal function of the intestinal epithelial barrier [23]. Thus, NLRP3 inflammasome activation in the IEC layer should help maintain homeostasis (Figure 1), whereas if the epithelial barrier is injured, inflammasome activation may have a harmful effect on sensing of commensal microbiota or bacterial clearance, hence making mucosal inflammation inevitable [24]. On the other hand, studies conducted on mice lacking *A*SC indicated an ameliorated inflammation, which could be correlated with ASC’s role as inflammasome activator or NF-kB pathway inhibitor [21,25]. Therefore, NLRP3-deficient mice could be protected against induced colitis, as a result of the reduced number of pro-inflammatory cytokines [26].

Besides the displayed side effects of NLRP3 inflammasome activation, another inflammasome has been shown to be associated with IBD, namely NLRP6. Studies on colitis induced-mice models lacking *Nlrp6* evidenced that IL-18 was less expressed [27], mucus secretion in goblet cells was dysregulated [28] and the overall clearance of bacterial pathogens was impaired, leading to alterations in the quantity and composition of the microbiota [24].

The two most important NLR proteins associated with IBD are NOD1 and NOD2, being crucial regulators of inflammatory responses to commensal microflora. 15–20% of IBD patients carry *Nod2* mutations, which account for alterations of intestinal immune homeostasis [29]. Despite the fact that UC and Crohn’s disease are related pathologies, there are differences in some of the susceptibility alleles. For example, if *Nod2* was one of the first genes to be associated with Crohn’s disease, only later on a studied developed by Freire et al. [30] correlated the *Nod2* mutations with a more aggravated condition for UC patients. Moreover, patients with *Nod2* mutations have been associated with defective Atg16L1 recruitment, leading to autophagy induction [31]. Therefore, NLRP3 inflammasome activation and single nucleotide polymorphism mutations in the *Nlrp3* have been widely reported to be correlated with pathogenesis and progression of IBD, including UC [14,19].

Although the exact mechanism that activates the NLRP3 inflammasome is unclear, it is well known that in quiescent cells, NLRP3 is associated with ER membranes [32]. When the cells are activated, NLRP3 is translocated to membranes positive for both ER and mitochondria. Thus, recent evidence also suggested that a K^+^ efflux and an increased ROS could be considered NLRP3 inflammasome activation stimuli [33].

## 3. Autophagy

Autophagy is a catabolic process of critical importance in cell and tissue homeostasis, as it regulates innate and adaptive immune system’s reaction, by controlling cytokine and inflammatory responses, as well as antigen presentation in immune cells [34]. Autophagy has been classified into three main types: chaperone-mediated autophagy, microautophagy, and macroautophagy, with macroautophagy being correlated with UC [35]. Macroautophagy, which, in the text, will be referred to as autophagy, is characterized by the formation of a double-membrane autophagosome, responsible for targeting and engulfing invading agents, damaged organelles and protein aggregates [36]. Later on, the autophagosome will fuse with the lysosome into an autolysosome, followed by degradation and removal of the substrates by lysosomal enzymes [37]. Assemble of the autophagosome and fusion with the lysosome are important steps controlled by a number of proteins, coded by autophagy-related (ATG) genes [38]. Although, until recently, *Atg16L1* mutants have only been associated and thoroughly investigated in the context of Crohn’s disease, new information regarding ATG16L1 implications in UC pathology started to be of interest for researchers. Thus, studies have indicated that some of the *Atg16L1* polymorphisms could be considered triggers for UC [39,40]. Moreover, along with the susceptibility to UC, patients with *Atg16L1* mutations are prone to a deficient mucosal healing [41].

Therefore, autophagy is vital for maintaining intracellular homeostasis, by recycling large protein complexes that cannot be degraded by the proteasome, complexes such as the active inflammasome [42].

### 3.1. Macroautophagy Controls NLRP3 Inflammasome Activation in Ulcerative Colitis Conditions

Recently, researchers have focused their attention towards the relationship between autophagy and NLRP3 inflammasome. Saitoh et al. [43] were the first to state that autophagy can both prevent and negatively control excessive NLRP3 inflammasome activation [38]. Their studies have shown that inhibiting autophagy, by loss or deficient production of autophagy proteins ATG16L1 and ATG7, results in increased CAS-1 cleavage and IL-1β and IL-18 release after inflammasome stimulation in MPs and DCs [43]. On the other hand, Dupont et al. [44] developed an autophagy model, which indicated that it could also be responsible for exocytosis of IL-1β, hence decreasing the accumulation of these high levels of IL-1β. Moreover, mice lacking *Atg5* or *Atg16L1* also displayed an increased IL-1β secretion [45]. Thus, patients with IBD might have a hyperactivity of the inflammasome in the absence of autophagy, generated by different mechanisms [32,46,47].

**Figure 2:**
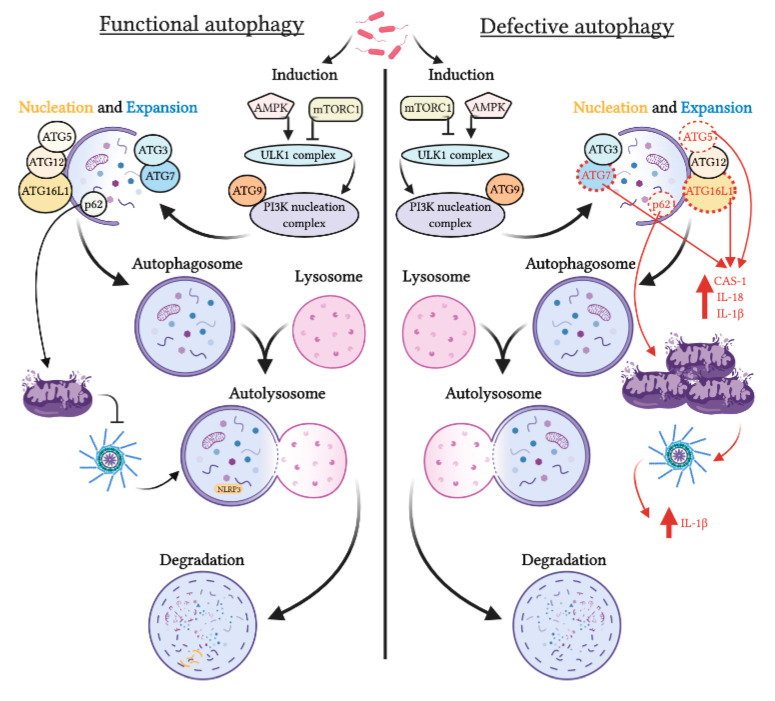
Comparison between functional and defective process of autophagy. Extracellular signals, such as pathogens, activate adenosine monophosphate-activated protein kinase (AMPK), which initiates ULK1 complex and PI3K nucleation complex assembly. PI3K complex recruits ATG proteins at the isolation membrane for its expansion and closing, forming the autophagosome. Adaptor protein p62 binds to organelles and protein complexes and brings them in the autophagosome. The autophagosome fuses with the lysosome to form the autolysosome and degrade the organelles using lysosomal enzymes. Under stress conditions, loss of ATG7 and Atg16L1 increases production of IL-18 and IL-1β. Moreover, when p62 is affected and damaged mitochondria accumulates, NLRP3 inflammasome is activated and IL-1β production increases, leading to inflammation.

The physical interaction between IBD risk factors Atg16L1, IRGM, and NOD2 has been recently validated, with IRGM helping microbes sensing, whereas Atg16L1 and NOD2 have antimicrobial defense properties [48]. Autophagy has been recently connected with NF-kB and mitogen-activated protein kinase (MAPK) signaling pathways, which are important regulators of the pro-inflammatory cytokines’ expression. IRGM can suppress these signaling pathways, hence negatively regulating IL-1β and IL-18 synthesis and cleavage, by downregulating NLRP3 inflammasome activation [15,48]. Moreover, NF-kB signaling pathway activates p62 expression, an adaptor protein responsible for the delivery of substrates to the autophagosome. p62 stimulates damaged mitochondria removal (Figure 2), thus inhibiting inflammasome activation and pro-inflammatory IL-1β synthesis [45,49]. In the absence of interaction with NLRP3 protein, inflammasome component ASC interacts with p62. The interaction between these two proteins suggests that autophagy could control aberrant inflammasome activation without inflammasome stimulation [42]. 

In contrast, studies developed by Dupont et al. identified a unique function of autophagy in yeast, where it could stimulate NLRP3 inflammasome activation, when cells are under starvation conditions [44,50].

Therefore, autophagy is a crucial element is maintaining NLRP3 inflammasome activation under control, by targeting and controlling the inflammasome components, such as NLRP3 protein, CAS-1 and pro-inflammatory cytokines IL-1β and IL-18 [51]. A well-balanced ratio between autophagy and inflammasome activation might be the key to a normal intestinal homeostasis for IBD patients [52].

### 3.2. Controlling Mitochondrial Damage and Mitochondrial ROS throughout Mitophagy in UC

There is no secret that a healthy mitochondrion is particularly important for a normal development of cellular processes. Numerous studies have registered that alterations to the epithelial cell mitochondria are an early event during inflammation, prior to tight junctions’ modifications [53,54]. Accordingly, studies developed by Rodenburg and colleagues on IECs have demonstrated that murine models with induced UC have a predisposition for abnormal mitochondria structure [34]. The abnormal structure might be due to the reduced levels of ATP within the intestine of UC patients. However, it is still to be postulated whether the damaged mitochondria appear as a consequence of pathogenesis of inflammation or the other way around [54,55].

When mitochondrion is injured, it starts releasing signals such as mitochondrial (mt) ROS, oxidized mtDNA or extracellular ATP efflux [56], which will be interpreted by the PRRs as DAMPs [57]. A study demonstrated that mtDNA released into the serum could represent a biomarker of inflammation for IBD patients [58].

Due to being responsible of removing the damaged organelles, including mitochondria, autophagy induces a decrease in the number of mitochondrial-derived DAMPs, leading to inflammasome activation suppression [38]. Besides the three aforementioned types of autophagy, there is a selective form of autophagy, mitophagy, used by the cells to maintain the health of mitochondria [59]. Mitophagy is activated when mitochondrial damage increases beyond a critical point, and it stimulates both the quality control mechanism of fission, which isolates the injured components of the depolarized mitochondrion, and also stops the reorganization of the damaged mitochondrion back into the network [54]. 

Damaged mitochondria result in elevated levels of ROS within the intestinal epithelium, which play a key role in intestinal inflammation occurrence and in DNA, proteins or lipids damage [59,60]. Therefore, mitophagy is responsible for suppression of NLRP3 inflammasome activation by removing injured mitochondria, hence limiting the release of ROS and mtDNA [61]. Since autophagy has also been correlated with a low level of ROS, loss of autophagy favors production of mtROS, which in turn enhances NLRP3 inflammasome activation [38,46]. Usually, normal levels of mtROS are kept under control by endogenous antioxidant scavengers. In addition, studies have indicated that under LPS stimulation, murine MPs deficient in anti-inflammatory cytokine IL-10 and mitophagy accumulate dysfunctional mitochondria. As dysfunctional mitochondria accumulate, ROS and mtDNA production increases in the cytosol, leading to excessive NLRP3 inflammasome activation [59,62]. Moreover, studies elaborated by Nishikawa et al. demonstrated that UC patients have a very high number of mtDNA mutations in the colon [63,64].

It has been demonstrated that in the absence of Atg16L1 autophagy protein, damaged mitochondria and accumulations of mtROS, respectively mtDNA, could further perpetuate the inflammation by altering the tight junction composition, which is responsible for the non-trespassing of bacteria into the lamina propria [58].

## 4. Endoplasmic Reticulum Stress and Unfolded Protein Response

IECs are exposed to a large number of factors derived from both the host and microbial environment, making them critical regulators of the immune response and microbiota [65]. In this manner, investigation of genes altered in the intestinal epithelium of IBD patients revealed another organelle that could be potentially associated with mucosal homeostasis and inflammation, the endoplasmic reticulum (ER) [66].

The ER is considered to act as a dynamic store, as it interacts with hormones and growth factors. ER regulates the biosynthesis of the proteins, as well as their assembly and folding. As a high number of unfolded and misfolded proteins accumulate in the ER lumen, the ER stress (ERS) emerges [67,68]. A protein is prone to unfolding or misfolding when changes in the non-covalent interactions may appear [69]. There is no clear evidence of why these proteins are not properly folded within the ER, but it is believed that either genetic or environmental factors could be the answer [8]. Among the factors that could trigger the incorrect protein folding, different studies have identified some forms of ROS that could have the strength to induce ERS [70]. Hence, an increased presence of oxidative stress and unfolded or misfolded proteins have been closely linked with ERS and IBD pathogenesis [71].

### 4.1. Endoplasmic Reticulum Unfolded Protein Response

ERS has been linked to IBD, because some of the cells’ function, such as goblet cells and Paneth cells, are dependent upon a normal ER. For the cells to cope with the stressful conditions caused by the increased amount of unfolded and misfolded proteins, they have developed the unfolded protein response (UPR) [8]. UPR is an adaptive signaling pathway, critical for the normal epithelial function and homeostasis, by targeting the abnormal function of the ERS [72]. Therefore, UPR facilitates the activation of pro-survival pathways, in order for cells to cope with stress or to initiate programed cell death [32].

UPR is maintained in an inactive state by being linked to a chaperone protein, glucose-regulated protein 78 (grp78), also called binding immunoglobulin protein (BiP). As unfolded and misfolded proteins accumulate, they start to bind to grp78, which results in activation of UPR. Once UPR is activated, the three main regulating transmembrane proteins of the UPR are released, respectively double-stranded RNA-dependent protein kinase (PKR)-like ER kinase (PERK), inositol-requiring 1α (IRE1) and activating transcription factor 6 (ATF6) [73]. 

The first response after UPR is activated emerges in dimerization and phosphorylation of PERK, leading to phosphorylation of elongation initiation factor 2α (eIF2α), consequently promoting inhibition of protein translation, except for a limited group of proteins, such as ATF4 [74,75]. Next, ATF6 migrates to the Golgi apparatus, where it undergoes proteolytic cleavage of its cytosolic tail, under the influence of site-1 and site-2 proteases. Consequently, the released fragment will translocate to the nucleus and activates transcription [76]. Last, but not least, IRE1, which has two isoforms, the ubiquitously expressed IRE1α and IRE1β, undergoes dimerization and autophosphorylation. Activated IRE1 possesses endoribonuclease activity and kinase activity, which activated Jun-related kinase (JNK) and NF-kB [66]. The IRE1 ribonuclease activity splices X-box binding protein-1 (*Xbp1*) [68]. 

When talking about a correlation between ERS, respectively UPR, and IBD, GWAS have identified several primary genetic factors affecting the UPR, which could be responsible for IBD pathogenesis. *Xbp1* is one of the susceptibility genes [8]. Loss of *Xbp1* has a tremendous impact upon the high sensitiveness of the epithelium in response to pro-inflammatory cytokines [66]. Another study that has focused on *Xbp1* as a key element for the development and maintenance of secretory cells [52,77]. Hence, *Xbp1* knockdown mice with induced colitis displayed an increased grp78 expression, as well as depletion of a large number of goblet and Paneth cells. As a result, *Xbp*1^−/−^ mice exhibited a lower response to pathogens interaction and a higher chance of developing inflammation [77,78].

### 4.2. Mitochondrial Unfolded Protein Response

Over time, cells have developed another stress-response mechanism in order to maintain homeostasis within the organelle, the mitochondrial UPR (UPRmt), activated during mitochondrial damage [54]. 

Under normal conditions, the stress activated transcription factor-1 (ATFS-1) is imported into the mitochondria and degraded. However, under mitochondrial stress, mitochondria efficiency is decreased and a small fraction of ATFS-1 accumulates in the cytosol. The UPRmt is regulated by ATFS-1, due to its nuclear localization sequence. ATFS-1 will be translocated to the nucleus, where it activates genes that stimulate protein folding, ROS decrease and protein import, suggesting that UPRmt’ could have the capability to restore mitochondrial homeostasis [79,80].

UPRmt in responsible for restoring homeostasis by increasing proteases and chaperones number [54,81]. Chaperones localized in the matrix are mandatory for protein import and a correct protein folding, whereas the matrix-localized proteases are required for degradation of incorrectly folded proteins. Disturbance of the mitochondrial biogenesis and protein import, as well as the presence of ROS can disrupt the mitochondrial protein-folding efficiency. Therefore, activation of the UPRmt increases the folding capability of the organelle during stress, restraining the incorrectly folded protein accumulation [82]. Although the UPRmt is activated to reestablish mitochondrial homeostasis, it can only react in order to enhance recovery of the mitochondria that is not totally damaged, whereas those who are beyond repair will be targeted for mitophagy [54].

## 5. Transcriptome Profiling of Non-Coding Regions in Genetic Susceptibility Loci

Researchers have come to the conclusion that a large number of SNPs could be associated with IBD pathogenesis, but the majority of these SNPs are located in the non-coding regions of the functionally responsible genes [83]. These non-coding elements can control gene expression, hence regulate the immune response and some of the biological activities. GWAS associated more than 200 genetic loci with either Crohn’s disease or UC, but most of the identified risk loci were shared between both of them [83,84]. These non-coding RNA (ncRNA) transcripts are represented by microRNA (miRNA) and long non-coding RNA (lncRNA) [84].

miRNAs are the most studied group of non-coding elements, due to their interaction with the translation process in the cytoplasm. Studies revealed that many miRNAs expression is altered in the mucosa of IBD patients [84]. Wu and colleagues [85] were the first group of researchers to identify the differentially expressed miRNAs in the mucosa of either active or inactive UC patients in comparison to healthy tissue (Table 1). For example, miR-192 and miR-422b have a significantly decreased expression in active tissues of UC patients compared to healthy control tissue. In contrast to the downregulated miRNAs, miR-21, mR-16, miR-24, miR-126, miR-23a and miR-29a were found to be upregulated [85]. Overexpressed miR-21 and miR-150 are responsible for an increase in intestinal epithelial permeability [86,87], whereas an increased expression of miR-126 was correlated with a decrease in IkBα, an inhibitor of the NF-kB pathway [88]. Altered expression of miR-145 and miR-212 impairs tight junctions’ function [89,90].

**Figure 3:**
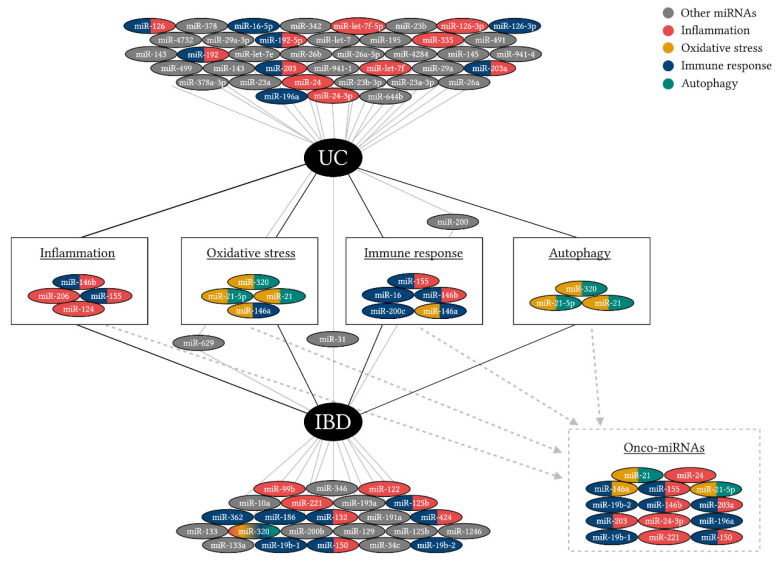
miRNAs correlation between UC and inflammatory bowel diseases (IBD), according to mirnet 1 network from miRNet. Up to this moment, there are 51 miRNAs known to be associated with UC pathology and 38 associated with IBD in general. This figure emphasizes the common miRNAs between UC and IBD in the case of inflammation, oxidative stress, immune response, and autophagy. Prolonged exposure to inflammation and oxidative stress could lead to tumorigenesis and colitis associated-colorectal cancer, based on the common miRNAs found in inflammation, immune response, oxidative stress, autophagy, and onco-miRNAs categories.

With the purpose to integrate miRNAs with the dysregulated processes aforementioned, an analysis was performed using miRNet, a miRNA network visual analytic tool (Figure 3). The unique miRNAs network for UC was identified by comparison with the non-coding RNAs representative for all types of IBD. The focus of this analysis was to emphasize and correlate miRNAs with their target and function in inflammation, immune response, autophagy, and oxidative stress. Moreover, miR-155 and miR-146b expression has been associated with inflammation and a defective immune response, whilst miR-21 and miR-21-5p altered expression has implications in the autophagy process, as well as in the oxidative stress. A prolonged state of inflammation in the UC patients’ colon could lead to colorectal cancer, known as colitis associated-colorectal cancer. This theory is strengthened/supported by the common miRNAs shared between inflammation, immune response, oxidative stress, and autophagy.

Whilst miRNAs have been thoroughly studied, little is known and understood about the implications of lncRNA in IBD. It is a widely held view that lncRNA could interact with gene regulation during transcription or epigenetic processes; hence, interfere in disease pathogenesis and progress. One of the first discovered lncRNA to be overexpressed in IBD patients, compared to healthy controls, was DQ786243 [91]. It upregulates the cyclic adenosine monophosphate response element-binding protein, leading to regulatory T cells dysregulation. Recent transcriptomic analysis have identified a number of 400 differentially expressed lncRNA exclusively associated with active or in remission UC [92]. Among these lncRNA molecules, interferon-gamma antisense RNA 1 (IFGN-AS1) was found to upregulate *Ifgn* expression in T cells, which encodes for an inflammatory cytokine. With an upregulated expression, the correlation between the inflammatory response in IBD and this lncRNA is undeniable [93]. Another UC specific lncRNA is BC012900 [94], which was found to be overexpressed by pathogens and cytokines through TLR and NLR pathways. The upregulated expression of BC012900 was associated with a reduction in cell proliferation and an increase in susceptibility to apoptosis. Besides the upregulated molecules, some lncRNAs were found to be downregulated in IBD biopsies, such as cyclin-dependent kinase inhibitor 2B AS1 (CDKN2B-AS1) [95], BC043570, HOXD-AS1, and phospholipase C delta 1 (PLCD1) [93].

There is growing evidence that some of these abnormally expressed miRNAs and lncRNAs could be considered potential biomarkers for IBD diagnosis, or even targets for treatment [83]. Consequently, future studies need to be developed in this area in order to broaden the potential horizons regarding the miRNA sequencing. Determining all of the upregulated and downregulated non-coding RNAs with a key role in IBD could increase the efficiency of diagnosis and therapeutic strategies [10].

## 6. Discussions

Among the well-known IBD, UC has some distinctive molecular features and characteristics. One of the most significant challenges in understanding the molecular mechanisms underlying UC pathogenesis is to have an integrative view and to correlate the particularities of UC-associated conditions- inflammation, inflammasome activation, oxidative stress and ROS generation, autophagy, ERS, UPR and the abnormal mucosal immune response. Despite the fact that, at first, these processes may seem to have nothing in common, thorough investigations have correlated impaired autophagy, as well as ERS with sudden intestinal inflammation [77]. Moreover, inflammasome activation is also dependent upon a normal autophagy, which plays a critical role in eradicating damaged mitochondria from the cytoplasm [46].

During UC, any alteration in the ER homeostasis leads to accumulation of misfolded proteins and further to UPR activation [96]. As unfolded proteins accumulate in the ER, a signal is transmitted to the nucleus in order to activate the UPR [97]. Patients with UC exhibited an improperly activated UPR, with IECs, especially Paneth cells and goblet cells, being highly affected by the ERS. In case of a defective UPR, IECs were observed to be having difficulty managing injury [98], as well as a lower renewal rate for the antimicrobial peptides-producing Paneth cells and a decreased number of mucin-producing goblet cells. Moreover, mice with loss of *Xbp1* function displayed complete absence of Paneth cells and a reduced number of goblet cells [8]. However, IECs alone cannot regulate intestinal homeostasis, but instead they respond to commensal microbiota and leukocyte populations stimuli [99,100]. When immune response is activated, leukocytes infiltrate within the intestinal epithelium, and under ERS, they adhere to the smooth muscle cells [101]. Another process that leukocytes depend on is the normal function of autophagy. Despite the fact that in the beginning the autophagy was considered to be a type of programmed cell death, the beliefs about it have changed over time and now its role is known to be of programmed cell survival [102].

Furthermore, it was shown that ER stress and dysregulated autophagy process act synergistically to promote UC development [45,103], following the observation that mice deficient for both *Atg16L1* and *Xbp1* developed an increased intestinal inflammation and a severe form of colitis. *Atg16L1*^−/−^ mice spontaneously developed intestinal inflammation [104], due to the increased ratio of pro-inflammatory cytokines [105]. Although it is not very well known either the autophagy process activates the ERS or the other way around, most of the studies support the first theory, which indicates that autophagy mediates the removal of the unfolded and misfolded proteins accumulated in the ER. To support this theory, recent studies associated the accumulation of a great number of unfolded and misfolded proteins in the ER, respectively an extended ERS, with the activation of the autophagy process. When ERS is prolonged, UPR promotes Ca^2+^ release from the ER, which activates the AMPK [106], leading to autophagy activation. After autophagy is activated and autophagosome fuses with the lysosome, it leads to removal of these aggregated proteins and help cells overcome the ERS [107]. Nevertheless, some of the UPR mediators, IRE1 [108] and PERK [109], can trigger autophagy activation via c-Jun N-terminal kinases, which causes autophagosome assemble [8]. In the absence of autophagy, cells are prone to ERS-induced death [108,110,111]. Regarding the implications that ERS could have in the perpetuation of the inflammation in the gut, Xue et al. [112] study indicated that ERS could be triggered by the effects caused by inflammation. For example, TNF-alfa, which is a pro-inflammatory cytokine, has been shown to be an aggravating factor for ERS.

There is a strong and dual link between the inflammatory background in the intestinal epithelium in UC and autophagy. For instance, decrease in Erbb2 interacting protein (ERBIN) expression in intestinal epithelium led to activation of autophagy and further to activation of colitis state [45,113]. Autophagy has also a direct impact on pro-inflammatory cytokine secretion, and is involved in regulating ROS levels [114]. Autophagy and ROS are involved in healthy IECs homeostasis and defense against pathogens. In order to prevent inflammation during UC, autophagy process is activated as a direct intracellular killing mechanism for pathogen degradation [47]. Furthermore, increased ROS production and cytokine secretion in UC intestinal epithelium was correlated with dysregulated inflammasome activation [62]. To prevent excessive inflammation, autophagy inhibits inflammasome formation. On the other hand, ROS and inflammasome are involved in regulating autophagy [45].

All these dysregulated UC-associated conditions developed on a highly inflammatory background are regulated at transcriptional, post-transcriptional, or post-translational levels by complex mechanisms. Among these, ncRNAs were found to be responsible for controlling these processes in a coordinated manner, thus resulting in common ncRNAs that can govern several interlinked inflammation-oxidative stress-autophagy-UPR states during UC. Several miRNAs were found to be dysregulated in UC, which have been only partially studied. This approach has to be further investigated to allow the identification of possible therapeutic targets for UC treatment.

An overlap of miRNAs found to be deregulated during UC and miRNAs with differential expression during general IBD was possible via a visual analytics platform and ncRNAs analysis tool called miRNet (Figure 3). The purpose of this analysis was to highlight the deregulated miRNAs common for several processes found to be abnormal during UC-associated inflammation, among them oxidative stress, autophagy and immune response, to stress-out the possibility of targeting these miRNAs by future therapeutic approaches in order to abolish the inflammatory status and associated responses in UC. We also wanted to highlight the particularities of UC-miRNAs profile compare to other IBDs and therefore we presented the common and distinct miRNAs features of UC as related to IBD (Figure 3). When performing this analysis, we found miR-124, miR-206, miR-155 and miR-146b to be common ncRNA markers for UC and IBD inflammation, among them miR-155 and miR-146b being also significant for the immune response. Interestingly, the same miRNAs (miR-320, miR-21, and miR-21-5p) are shared markers for oxidative stress and autophagy related to both UC and IBD, while miR-146a links oxidative stress and immune response. Finding these commonly deregulated miRNAs strengthens the previously discussed interlink between oxidative stress and autophagy in UC-inflammation conditions. An overly accumulation of mtROS, due to deficient autophagy, could induce oxidative stress [115,116]. Therefore, as mentioned before, the aberrant activation of the inflammasome is controlled by the autophagy process, an interplay between autolysosome formation and oxidative stress leads to the idea that oxidative stress could also contribute to inflammasome activation [117].

Even more interesting, when comparing miRNAs profiles during UC and IBD, we found a set of miRNAs, which are common for inflammation, oxidative stress, autophagy, and immune response and that hypothetically link these inflammatory-associated states with oncogenesis. Previous studies have shown that miRNAs can mediate crosstalk between UC and colorectal cancer or colitis-associated cancer [118,119]. For example, miR-125b and miR-155 were found upregulated in the inflamed mucosa, controlling genes involved in the inflammatory pathways [119,120], while also being dysregulated in colorectal cancer [119,121]. These miRNAs, along with miR-138, mir-223, miR-200a, and miR-378 were found upregulated in the inflamed colonic mucosa of UC patients [119]. In our analysis, miR-21, miR-24, miR-155, miR-146b, miR-203, miR-221, and miR-150 were identified as onco-miRNAs (Figure 3); thus, supporting the possibility that the chronic inflammation during UC facilitates the background for oncogenesis.

## 7. Conclusions

UPR and autophagy pathways have a key role in maintaining the intestinal homeostasis. UC is characterized by a chronic inflammatory state of the intestinal mucosa, associated with dysregulated autophagy and UPR. Imbalance in these processes and its consequences upon UC development and progression needs to be further investigated and the molecular mechanisms involved need to be unveiled or confirmed. Accumulating evidence has recently correlated the abnormal expression of non-coding regions of the genome with diseases whose causes are represented by complex interactions between different mechanisms. Although some of the miRNAs and lncRNAs functions in the IBD have been established, the majority of them have not been yet clarified. This review highlights the overlapping miRNAs specific for inflammation, oxidative stress, and autophagy in UC with the ones found deregulated in IBD, pointing out also the differences in the post-transcriptional control of these pathologies. Moreover, there are some miRNA species found to be upregulated in UC and also in colitis associated cancer, thus supporting the possibility that in certain conditions, the chronic inflammatory background, dysregulated UPR and autophagy characteristic in UC might lead to tumorigenesis.

To conclude, further studies need to thoroughly evaluate the implications of ncRNAs in UC, as well as to completely decipher the correlation between inflammatory state, autophagy, and ER stress, for a better understanding of UC as part of IBD and development of new strategies for prevention and treatment.

## Figures and Tables

**Table 1:** Most frequently upregulated and downregulated miRNAs in active or inactive ulcerative colitis (UC) samples (vs. = versus).

microRNA	Expression	Type of Disease	Sample Type
miR-16	Upregulated	Active UC vs. *control*	Sigmoid colon biopsies
miR-21	Upregulated	Active UC vs. *control*	Sigmoid colon biopsies
miR-23a	Upregulated	Active/Inactive UC vs. *control*	Sigmoid colon biopsies
miR-24	Upregulated	Active UC vs. *control*	Sigmoid colon biopsies
miR-29a	Upregulated	Active/Inactive UC vs. *control*	Sigmoid colon biopsies
miR-126	Upregulated	Active UC vs. *control*	Sigmoid colon biopsies
miR-138	Upregulated	Active UC vs. *inactive UC*	Colon biopsies
miR-150	Upregulated	Active UC vs. *inactive UC*	Colon biopsies
miR-192	Downregulated	Active UC vs. *control*	Peripheral blood
miR-212	Downregulated	Inactive UC vs. *control*	Colon biopsies
miR-375	Downregulated	Active UC vs. *control*	Colon biopsies
miR-422b	Upregulated	Active UC vs. *control*	Peripheral blood
